# Letter to the Editor regarding: “Pre-and post-analytical guidelines for the microscopic diagnosis of melanoma: recommendations from the Brazilian Society of Pathology”^☆^

**DOI:** 10.1016/j.abd.2025.501271

**Published:** 2026-01-09

**Authors:** José Antonio Jabur da Cunha, Roberto Gomes Tarle, Glaysson Tassara Tavares

**Affiliations:** a Clinic of Dermatology, Irmandade da Santa Casa de Misericórdia de São Paulo, São Paulo, SP, Brazil; b Escola de Medicina, Pontifícia Universidade Católica do Paraná, Curitiba, PR, Brazil; c Hospital das Clínicas, Universidade Federal de Minas Gerais, Belo Horizonte, MG, Brazil

Dear Editor,

We would like to express some concerns regarding the conclusions of Xavier-Junior et al.^1^ The authors stated that “the use of Mohs Micrographic Surgery (MMS) is not validated for the management of melanocytic lesions and may compromise diagnostic accuracy”, referencing a study that evaluated bias in trials involving both MMS and Staged Excision (SE).^2^

In that study, the authors concluded that randomized, bias-controlled trials comparing Wide Local Excision (WLE), SE, and MMS are needed, without asserting that MMS or SE are invalid. Furthermore, the study made no reference to WLE research, including those addressing lesions on the head and neck, which might have contributed to a more technically informed comparison among techniques.

The authors also observed that freezing a tumor suspected of melanoma may compromise diagnostic accuracy. One referenced study mentioned two investigations. The first discouraged the use of frozen sections for prognostic parameters ‒ a point with which we agree. The diagnosis and microstaging of melanoma should be performed on permanent paraffin-embedded sections. It is important to note, however, that the same study highlighted that, in specific situations, MMS may offer advantages in the surgical management of melanoma.

The second referenced study^3^ compared margin assessment in frozen en face sections of 13 melanomas with subsequent paraffin sections. However, the small and heterogeneous sample, lack of technical standardization, and the flawed assumption that deeper paraffin sections represent the gold standard severely limit its conclusions. The study measures sampling variability rather than true diagnostic accuracy or recurrence risk. It is known that 4 μm sections and rapid-freezing techniques are required for optimal histologic quality.

The National Comprehensive Cancer Network recommends MMS as an option for minimally invasive melanomas located in anatomically constrained areas. A recent systematic review and meta-analysis demonstrated higher cure rates than those achieved with WLE, with significantly lower local recurrence.^4^ In addition, MMS has been associated with improved overall survival in head and neck melanomas.^5^

In special situations ‒ such as large, ill-defined lesions located in anatomically constrained areas ‒ techniques that allow complete margin assessment are crucial. In such cases, traditional histologic processing methods that assess only a portion of the true surgical margin raise concerns about incomplete excision and recurrence.

Despite ongoing criticism regarding the challenges of frozen-section analysis in melanocytic lesions, margin evaluation during MMS has proven effective, is widely validated in the literature, and is endorsed by leading international oncology panels.

## ORCID IDs

Roberto Gomes Tarlé: 0000-0003-2831-6579

Glaysson Tassara Tavares: 0000-0002-1688-2955

## Authors’ contributions

José Antonio Jabur da Cunha: Critical literature review; manuscript critical review; preparation and writing of the manuscript; study conception and planning.

Roberto Gomes Tarlé: Critical literature review; manuscript critical review; preparation and writing of the manuscript; study conception and planning.

Glaysson Tassara Tavares: Critical literature review; manuscript critical review; preparation and writing of the manuscript; study conception and planning.

## Financial support

None declared.

## Research data availability

Does not apply.

## Conflicts of interest

None declared.
